# Primary repair of esophageal perforation: Case report

**DOI:** 10.1016/j.ijscr.2020.04.026

**Published:** 2020-05-11

**Authors:** Akello W. Abila, Mburu E. Nditika, Rono D. Kipkemoi, Stephen Ondigo, Barasa O. Khwa-Otsyula

**Affiliations:** a Department of Surgery and Anesthesiology, Moi University, School of Medicine, Eldoret, Kenya; b Faculty of Health Sciences, Egerton University, Nakuru, Kenya; c Department of Cardiothoracic Surgery, Moi Teaching and Referral Hospital, Eldoret, Kenya

**Keywords:** Esophageal perforation, Primary repair, Esophageal injury, Case report

## Abstract

**Introduction:**

Mortality after esophageal perforation is high irrespective of the treatment modality. The rarity of traumatic esophageal perforations has made it difficult to conduct comprehensive studies that can answer pertinent questions with regard to management.

**Presentation of case:**

We report a case of through and through thoracic esophageal injury caused by an assailant’s arrow in a young physically active male adult. Diagnosis was made on-table. He successfully underwent primary repair of the esophageal injury 16 h post injury via a left thoracotomy. Recurrent lung collapse and pleural effusion was managed with tube thoracostomy and chest physiotherapy.

**Discussion:**

Esophageal perforations occur infrequently and may produce vague symptoms leading to diagnostic and therapeutic delays. High index of suspicion particularly in penetrating chest trauma followed by relevant investigations may reduce delay. Principles of management include treatment of contamination, wide local drainage, source control and nutritional support. Source control is achieved surgically or through endoluminal placement of stents. Surgical options include primary repair, creation of a controlled fistula by T-tube or esophageal exclusion.

**Conclusion:**

Primary repair of traumatic injury to a healthy esophagus is feasible for cases diagnosed early and without significant mediastinal contamination as in our case. Associated injuries are more likely in such cases to lead to increased morbidity and prolonged hospital stay and must be handled carefully.

## Highlights

• Most common mechanism of esophageal perforation is iatrogenic.
• High index of suspicion in penetrating chest trauma followed by relevant investigations may reduce delay.
• Early primary repair is sufficient for select cases of traumatic esophageal perforation.
• Associated injuries are more likely in such cases to lead to increased morbidity.

## 1 Introduction

Mortality after esophageal perforation is high irrespective of the modality of treatment [[1], [2], [3], [4]]. Esophageal injuries also carry a high morbidity and often result in increased duration of hospitalization [[5], [6], [7]]. Different operative and non-operative approaches to treatment have been reported with variable outcomes. It is also not clear what factors determine successful management [1,8,9]. The rarity of traumatic esophageal perforations does not allow comprehensive studies to answer important questions regarding management.

This case report has been reported in line with the SCARE criteria [10]. The patient was managed at Moi Teaching and Referral Hospital (MTRH), which is a public teaching and referral hospital with subspecialists in various disciplines.

## 2 Case

**Fig. 1:**
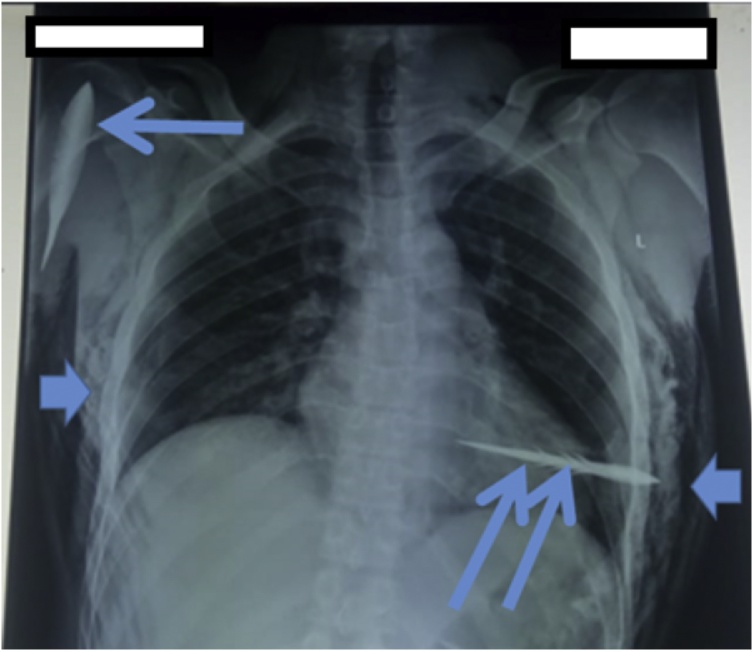
Chest radiograph of the patient; Single long arrow - arrow head superimposed on humeral head; Two long arrows - Arrow superimposed on cardiac silhouette and lung field; Short arrows - emphysema over right and lateral chest walls.

30 year old male was shot by an arrow which went through the 4th intercostal space just behind anterior axillary fold, penetrated the right chest wall to enter the chest cavity and lodged in the left thoracic wall. A second arrow entered the axilla from the posterior aspect of the upper part of the right arm. Pressure dressings had been applied around the entry points of the arrows. There was no obvious active bleeding externally. A chest radiograph done at the referring facility showed an arrow traversing both chest cavities superimposed on the cardiac silhouette (Fig. 1). The second arrow had its tip superimposed on the humeral head.

Patient was referred to our facility, Moi teaching and referral hospital (MTRH) from a peripheral facility. He received a unit of packed red blood cells while on transit, intravenous fluids, and oxygen. A dose of analgesic, antibiotics and tetanus toxoid had been administered as per the referral note. Patient’s past medical history was not significant for chronic illness or prior surgeries, and had no reported drug or food allergies. He did not smoke or use alcohol. He lived a very active life working on his family farm mostly by hand.

At the MTRH emergency department, initial vitals were blood pressure 90/54 mmHg, pulse rate 90, SPO_2_ 91% in room air, respiratory rate 22 and temperature 36.6 °C. Patient was fully conscious and complained of chest pain. Patient had equal bilateral chest expansion and air entry but with extensive emphysema of the left more than right chest wall. His blood pressures stabilized following resuscitation with crystalloids and dextran (Blood products were not immediately available). Analgesics and antibiotics were continued as per the treatment sheet. Renal function tests were normal and hemoglobin was 12 g/dL (12.0–17.4 g/dL) with platelet count of 187 × 10^3^/μL (150–400 × 10^3^/μL) and white blood cells (WBC) of 13.23 × 10^3^/μL (5.00–10.00 × 10^3^/μL). He was prepared and wheeled to theatre stable about 15 h post injury. The lead was a cardiothoracic surgeon.

Intra-operatively, the right axillary injury was dealt with first as there was active bleeding after removal of pressure dressing. A delto-pectoral groove incision was used to access the axilla and a distal approach through floor of axilla was employed. The severed right axillary vein was identified and ligated. The right axillary artery was not injured. The arrow head was identified, removed. Incision was then closed.

Patient was repositioned for a left thoracotomy, about 16 h post injury. Intra-op findings were 2 clean through and through cuts of the anterior and posterior wall of the thoracic esophagus, and a left hemothorax of about 500 mL. The arrow had gone through the left lung to lodge into the left thoracic wall. There were no injuries to the aorta and heart.

Arrow was gently pulled out. The esophagus was mobilized minimally at the level of injury. Minimal debridement was done; the esophageal tissue was healthy without edema. There was no visible soiling around the injury. A nasogastric tube (NGT) was passed into the stomach. Both posterior and anterior perforations were repaired from outside in two layers with Silk over the NGT. Volume of fluid suctioned from the left chest cavity was about 500 mL which seemed like a mixture of blood and saliva. Irrigation and lavage of the pleural cavity was done, left chest tube inserted and thoracotomy incision closed in layers. Patient was then repositioned to supine and a right chest tube inserted. It was deemed prudent to insert a prophylactic right chest tube as the arrow had traversed both chest cavities. Patient was transfused with 1 unit of packed red blood cells intra-operatively. Unfortunately the NGT dislodged at the time of reversal from anesthesia and the patient was nursed without it.

Post-operative plan included nil per oral, intravenous fluids, analgesics, antibiotics and proton pump inhibitors (PPIs). On the 6^th^ post-op day the left chest tube was removed. A contrast swallow studies done on day 8 showed no leak at site of anastomosis with contrast in the stomach. Subsequently patient was started on oral sips. On day 10, patient developed respiratory distress necessitating an emergency chest radiograph which showed left lung collapse without effusion. Left lung expanded on chest physiotherapy and adequate analgesia.

A chest radiograph done on day 14 when patient went into distress again requiring oxygen showed a right pleural effusion with the chest tube insitu. The chest tube was removed and another fixed which drained serosanguinous fluid without food contents. Over next several days, patient continued to improve and the chest tube was removed. The patient was ambulant, doing self-care, off antibiotics and feeding normally. He was discharged on post-operative day 25 for follow up at the specialist clinic. Patient was well and feeding comfortably when he was seen at the outpatient clinic 3 weeks after discharge.

## 3 Discussion

In our case the patient factors were favorable; a previously healthy 30 year old physically active male who was not a smoker. Esophageal perforation was caused by low energy missiles resulting in 2 clean through and through cuts of the wall, with minimal loss of tissue. Although patient manifested early features of shock, resuscitation maintained the patient stable, and he had been started on broad-spectrum antibiotics and analgesics within a short time of injury. Diagnosis was made early on-table at exploration and esophageal repair was done at the 16^th^ hour. There was also minimal soiling of the mediastinum. Contrast swallow studies done on the 8^th^ post-operative day showed no anastomotic leak with contrast in the stomach. There was no pooling of contrast at the level of repair. Subsequently the patient was able to feed well and comfortably. Notably, the patient had prolonged hospital stay of 25 days. The additional 18 days were due to complications from other chest injuries.

Esophageal perforations are transmural disruptions of the esophagus that subsequently lead to leakage of intraluminal contents into the surrounding mediastinum. This causes local inflammation, a systemic inflammatory response, and eventually the development of sepsis that results in significant morbidity and mortality [[1], [2], [3], [4]].

Overall, the most common mechanism of esophageal perforation is iatrogenic following endoscopic and other surgical procedures [[1], [2], [3],^5^,^11^]. Traumatic mechanisms may be either blunt (e.g., motor vehicle crash) or penetrating (e.g., gunshot or stab wounds). Other mechanisms include foreign bodies, spontaneous rupture (e.g., Boerhaave syndrome), and ingestion of acid or caustic substances.

Esophageal perforations occur infrequently and may produce vague symptoms leading to diagnostic and therapeutic delays [1,5,11]. Esophageal injury should be suspected in the predisposed patient with symptoms of epigastric or chest pain, neck or throat pain, and dysphagia. Physical examination findings might include crepitus on the chest, neck, or face; neck swelling; epigastric tenderness; nasal voice; or sometimes normal examination findings. Other early evidence might include a chest radiograph with mediastinal emphysema, free intra-abdominal air, or pleural effusion. The mechanism of injury can be the greatest clue that would initiate further workup. High index of suspicion particularly in penetrating chest trauma followed by relevant investigations may reduce delay.

Diagnostic procedures include endoscopy, computed tomography (CT) and a gastrografin esophagography. Gastrograffin esophagography is the study of choice in suspected esophageal perforations [5,8]. When gastrograffin study is negative despite suspicion of a perforation, CT scan with contrast which has the lowest false negative rate of 1% comes in handy in making a diagnosis, [11]. A Gastrograffin study has a false negative rate of 10% and carries the risk of chemical pneumonitis. Endoscopy carries the advantage of direct visualization, assessment of the size of perforation and the viability of surrounding epithelium, and therefore doubles as a diagnostic management planning tool. However, endoscopy carries the risk of enlarging the perforation. In our case as indicated earlier, the diagnosis was made on-table during emergency thoracotomy.

The principles of management after diagnosis of a perforation include treatment of contamination, wide local drainage, source control and nutritional support. Drainage of the area is achieved using chest tubes or imaging guided drains. Extensive leaks may require thoracotomy with decortication or video assisted thoracoscopy (VATS) where available. Source control is achieved surgically or through endoluminal placement of self-expandable metallic stents (SEMs). Surgical options include primary repair, creation of a controlled fistula by T-tube or esophageal exclusion in cases of large tissue defects. Approach to management may be conservative or more aggressive depending on various factors.

Conservative treatment with cessation of oral intake, broad-spectrum antibiotics, including antifungals and parenteral nutritional support or enteral feeding access, is feasible for iatrogenic perforations with minimal mediastinal soiling [8]. This management is continued for 7 days and thereafter followed by contrast esophagogram till leakage resolves so long as the patient demonstrates improvement. In cervical perforations with no sign of mediastinal contamination conservative approach or drainage only may be sufficient initially [8,11].

The acute surgical treatment of esophageal perforation is aimed at sealing the perforation and drainage of the mediastinum to prevent sepsis [1,2,5,8,11]. Surgical approaches depend on the level of perforation where high perforations are approached through a left-sided neck incision, mid-esophageal through a right thoracotomy, and distal esophageal through a left thoracolaparatomy.

Primary repair in two layers with or without reinforcement is the treatment of choice for small perforations with healthy tissues [6,8,12,13]. Several groups report good results with this technique even in patients who come to surgery late [11]. However, in the recent past some experts advocated that primary repair should only be used in patients with early perforations and recommend resection or diversion when the perforation is older than 24 h.

With severe contamination of the pleural cavity as occurs in spontaneous perforations of the distal esophagus and in failed conservative management then operative management is recommended with drainage, debridement and closure where feasible [2,5]. Extensive injuries with devitalized areas can be managed with controlled fistula using a T-tube. Very large defects warrant esophageal exclusion with creation of a cervical esophagostomy and gastrostomy tube, with plans for future esophagectomy and reconstruction with gastric, colon, or small bowel conduit [5,14]. In cases of malignant disease in the esophagus a resection is recommended.

There is growing use of self-expanding stents in benign esophageal perforation. For small iatrogenic esophageal perforations, Self-Expandable Metallic Stents (SEMS) in combination with thoracic tubes drainage have been used successfully [15,16]. SEMS migration and difficult removal are however challenges in distal esophagus as well as strictures with metallic stents [16].

Mortality rate associated with esophageal perforations ranges between 10% and 40% and may depend on a variety of factors, including the cause of the perforation, the presence of any underlying esophageal pathology, the location of the perforation, diagnostic or treatment delay, the method of treatment employed, comorbid conditions and the extent of the injury [2,3,5,16]. 2 series showed that only time to diagnosis had an influence on mortality [1,8]. A Swedish study showed the single most important factor influencing mortality was the pre-op American Society of Anaesthesiologists (ASA) status [11].

For patients who survive esophageal perforations, high levels of morbidity, long hospital and intensive care unit (ICU) stays have been reported [[5], [6], [7]]. In our case the patient had prolonged hospitalization mainly due to associated chest injuries.

The Pittsburgh group has suggested a perforation severity score (PSS) for better decision making in the management of esophageal perforation [17]. The perforation severity score (PSS) is designed to measure the seriousness of esophageal disruption by weighting clinical variables, which are possible indicators of injury severity and patient outcome. The score has been shown to predict morbidity and mortality in select patient subgroups [18].

## 4 Conclusion

Primary repair of traumatic injury to a healthy esophagus is feasible, especially for cases diagnosed early and without significant mediastinal contamination as in our case. Associated injuries are more likely in such cases to lead to increased morbidity and prolonged hospital stay and must be handled carefully.

## Declaration of competing interest

None.

## Sources of funding

No funds raised for this case report.

## Ethical approval

The proposal to write the case report was submitted alongside the signed consent forms to the Moi University/Moi Teaching and Referral hospital Institutional Research and Ethics Committee (IREC) and approved.

## Consent

Informed consent was obtained from the patient. No identifying information has been published.

## Author contribution

**Akello W. Abila:** Conceptualization, Methodology, Writing – Original draft preparation, Project administration.

**Mburu E. Nditika:** Conceptualization, Methodology, Writing - Original draft preparation.

**Rono D. Kipkemoi:** Conceptualization, Methodology, Writing - Original draft preparation.

**Stephen Ondigo:** Methodology, Writing – Reviewing and Editing.

**Barasa O. K. Otsyula:** Conceptualization, Methodology, Writing-Reviewing and Editing, Supervision.

Chacha C. Mwita: contributor.

## Registration of research studies

NA.

## Guarantor

Akello W. Abila.

Mburu E. Nditika.

Rono D. Kipkemoi.

Stephen Ondigo.

Barasa O. K. Otsyula.

## Statement on patient details

We hereby confirm that the figures in the manuscript do not include any patient details.

## Ethical considerations

The case report was carried out after ethical approval by the Institutional Research and Ethics Committee (IREC) of MTRH and Moi University School of Medicine. In addition, administrative approval was sought from the MRTH management. Informed consent to participate in the study was obtained from the participant.

## Provenance and peer review

Editorially reviewed, not externally peer-reviewed.

## References

[1] Muir A., White J., McGuigan J., McManus K., Graham A. (2003). Treatment and outcomes of oesophageal perforation in a tertiary referral centre. Eur. J. Cardio-Thoracic Surg..

[2] Nirula R. (2013). Esophageal perforation. Surg. Clin. North Am..

[3] Mavroudis C.D., Kucharczuk J.C. (2014). Acute management of esophageal perforation. Curr. Surg. Rep..

[4] Mubang R.N., Stawicki S.P. (2018). Trauma, Esophageal.

[5] Kaman L., Iqbal J., Kundil B., Kochhar R. (2010). Management of esophageal perforation in adults. Gastroenterol. Res..

[6] Zimmermann M., Hoffmann M., Jungbluth T., Bruch H.P., Keck T., Schloericke E., Zimmermann M. (2016). Predictors of morbidity and mortality in esophageal perforation: retrospective study of 80 patients. Scand. J. Surg..

[7] Andrade-Alegre R. (2005). Surgical treatment of traumatic esophageal perforations: analysis of 10 cases. Clinics.

[8] Vallböhmer D., Hölscher A.H., Hölscher M., Bludau M., Gutschow C., Stippel D., Bollschweiler E., Schröder W. (2010). Options in the management of esophageal perforation: analysis over a 12-year period. Dis. Esophagus.

[9] Hermansson M., Johansson J., Gudbjartsson T., Hambreus G., Jönsson P., Lillo-Gil R., Smedh U., Zilling T. (2010). Esophageal perforation in South of Sweden: results of surgical treatment in 125 consecutive patients. BMC Surg..

[10] Agha R.A., Borrelli M.R., Farwana R., Koshy K., Fowler A.J., Orgill D.P., Zhu H., Alsawadi A., Noureldin A., Rao A., Enam A., Thoma A., Bashashati M., Vasudevan B., Beamish A., Challacombe B., De Wilde R.L., Machado-Aranda D., Laskin D., Muzumdar D., D’cruz A., Manning T., Healy D., Pagano D., Goel P., Ranganathan P., Pai P.S., Raja S., Ather M.H., Kadioäžlu H., Nixon I., Mukherjee I., Gómez Rivas J., Raveendran K., Derbyshire L., Valmasoni M., Chalkoo M., Raison N., Muensterer O., Bradley P., Roberto C., Afifi R., Rosin D., Klappenbach R., Wynn R., Giordano S., Basu S., Surani S., Suman P., Thorat M., Kasi V. (2018). The SCARE 2018 statement: Updating consensus Surgical CAse REport (SCARE) guidelines. Int. J. Surg..

[11] Hermansson M., Johansson J., Gudbjartsson T., Hambreus G., Jönsson P., Lillo-gil R., Smedh U., Zilling T. (2010). Esophageal perforation in south of Sweden: results of surgical treatment in 125 consecutive patients. BMC Surg..

[12] Sung S.W., Park J., Kim Y.T., Kim J.H. (2002). Surgery in thoracic esophageal perforation: primary repair is feasible. Dis. Esophagus.

[13] Nishimaki T., Ono K., Tada T., Hatakeyama K. (2001). Successful primary reinforced repair of esophageal perforation using a pedicled omental graft through a transhiatal approach. Dis. Esophagus.

[14] Linden P.A., Bueno R., Mentzer S.J., Zellos L., Lebenthal A., Colson Y.L., Sugarbaker D.J., Jaklitsch M.T. (2007). Modified T-tube repair of delayed esophageal perforation results in a low mortality rate similar to that seen with acute perforations. Ann. Thorac. Surg..

[15] Sudarshan M., Elharram M., Spicer J. (2016). Management of esophageal perforation in the endoscopic era: is operative repair still relevant?. Surgery.

[16] Dasari M., Neely D., Kennedy A., Spence G., Rice P., Mackle E., Epanomeritakis E. (2014). The role of esophageal stents in the management of esophageal anastomotic leaks and benign esophageal perforations. Ann. Surg..

[17] Abbas G., Schuchert M.J., Pettiford B.L., Pennathur A., Landreneau J., Landreneau J., Luketich J.D., Landreneau R.J. (2009). Contemporaneous management of esophageal perforation. Surgery.

[18] Schweigert M., Santos Sousa H., Solymosi N., Yankulov A., Fernández M.J., Beattie R., Dubecz A., Rabl C., Law S., Tong D., Petrov D., Schäbitz A., Stadlhuber R.J., Gumpp J., Ofner D., McGuigan J., Costa-Maia J., Witzigmann H., Stein H.J. (2016). Spotlight on esophageal perforation: a multinational study using the Pittsburgh esophageal perforation severity scoring system. J. Thorac. Cardiovasc. Surg..

